# Author Correction: Detection of COVID-19 using multimodal data from a wearable device: results from the first TemPredict Study

**DOI:** 10.1038/s41598-022-08723-x

**Published:** 2022-03-16

**Authors:** Ashley E. Mason, Frederick M. Hecht, Shakti K. Davis, Joseph L. Natale, Wendy Hartogensis, Natalie Damaso, Kajal T. Claypool, Stephan Dilchert, Subhasis Dasgupta, Shweta Purawat, Varun K. Viswanath, Amit Klein, Anoushka Chowdhary, Sarah M. Fisher, Claudine Anglo, Karena Y. Puldon, Danou Veasna, Jenifer G. Prather, Leena S. Pandya, Lindsey M. Fox, Michael Busch, Casey Giordano, Brittany K. Mercado, Jining Song, Rafael Jaimes, Brian S. Baum, Brian A. Telfer, Casandra W. Philipson, Paula P. Collins, Adam A. Rao, Edward J. Wang, Rachel H. Bandi, Bianca J. Choe, Elissa S. Epel, Stephen K. Epstein, Joanne B. Krasnoff, Marco B. Lee, Shi-Wen Lee, Gina M. Lopez, Arpan Mehta, Laura D. Melville, Tiffany S. Moon, Lilianne R. Mujica-Parodi, Kimberly M. Noel, Michael A. Orosco, Jesse M. Rideout, Janet D. Robishaw, Robert M. Rodriguez, Kaushal H. Shah, Jonathan H. Siegal, Amarnath Gupta, Ilkay Altintas, Benjamin L. Smarr

**Affiliations:** 1 Osher Center for Integrative Health, University of California San Francisco, San Francisco, CA USA; 2 MIT Lincoln Laboratory, Massachusetts Institute of Technology, Lexington, MA USA; 3 Halıcıoğlu Data Science Institute, University of California San Diego, La Jolla, CA USA; 4 Department of Biomedical Informatics, Harvard Medical School, Boston, MA USA; 5 Department of Management, Zicklin School of Business, Baruch College, The City University of New York, New York, NY USA; 6 San Diego Supercomputer Center, University of California San Diego, San Diego, CA USA; 7 Department of Electrical and Computer Engineering, University of California San Diego, La Jolla, CA USA; 8 Department of Bioengineering: Bioinformatics, University of California San Diego, San Diego, CA USA; 9 Department of Psychology, Drexel University, Pennsylvania, PA USA; 10 Vitalant Research Institute, University of California San Francisco, San Francisco, CA USA; 11 Department of Psychology, University of Minnesota - Twin Cities, Minneapolis, MN USA; 12 Love School of Business, Elon University, Elon, NC USA; 13 School of Medicine, University of California San Francisco, San Francisco, CA USA; 14 Department of Anesthesiology, Northwestern McGaw Medical Center, Feinberg School of Medicine, Chicago, IL USA; 15 Department of Emergency Medicine, University of California Los Angeles Health, Los Angeles, CA USA; 16 Center for Health and Community, University of California San Francisco, San Francisco, CA USA; 17 Department of Emergency Medicine, Beth Israel Deaconess Medical Center Boston, Boston, MA USA; 18 Schmidt College of Medicine, Florida Atlantic University, Boca Raton, FL USA; 19 Department of Neurosurgery, Santa Clara Valley Medical Center, Stanford University, San Jose, CA USA; 20 Department of Emergency Medicine, Jamaica Hospital Medical Center, Jamaica, NY USA; 21 Department of Emergency Medicine, Boston Medical Center, Boston, MA USA; 22 Department of Anesthesiology: Pain Management and Perioperative Medicine, University of Miami, Miami, FL USA; 23 Department of Emergency Medicine, New York Presbyterian Brooklyn Methodist Hospital, Brooklyn, NY USA; 24 Department of Anesthesiology and Pain Management, University of Texas Southwestern, Dallas, TX USA; 25 Department of Biomedical Engineering, Renaissance School of Medicine, Stony Brook University, Stony Brook, NY USA; 26 Stony Brook Medicine, Stony Brook University Renaissance School of Medicine, Stony Brook, NY USA; 27 Department of Anesthesia: Perioperative and Pain Medicine, Kaiser Permanente San Diego, San Diego, CA USA; 28 Department of Emergency Medicine, Tufts Medical Center, Boston, MA USA; 29 Department of Emergency Medicine, University of California San Francisco, San Francisco, CA USA; 30 Weill Cornell Medical Center, Weill Cornell Medical School, New York, NY USA; 31 New York Presbyterian Queens, Weill-Cornell Medical College, Queens, NY USA

Correction to: *Scientific Reports* 10.1038/s41598-022-07314-0, published online 02 March 2022

The original version of this Article contained errors in the Funding section.

“The USAMRDC under the Department of Defense (DOD) provided financial support for this work. This effort was funded under MTEC solicitation MTEC-20-12-Diagnostics-023 and is funded by the USAMRDC under the Department of Defense. The views and conclusions contained herein are those of the authors and should not be interpreted as necessarily representing the official policies or endorsements, either expressed or implied, of the U.S. Government. The #StartSmall foundation provided financial support for this work. The US Department of Defense Air Force Office of Scientific Research, through the Massachusetts Institute of Technology Lincoln Laboratory (MIT-LL), provided financial support for this work. Oura Health Oy provided 1400 pieces of hardware and financial support in the form of a sponsored research contract.”

now reads:

“The USAMRDC under the Department of Defense (DOD) provided financial support for this work. This effort was funded under MTEC solicitation MTEC-20-12-Diagnostics-023 and is funded by the USAMRDC under the Department of Defense. This effort is also based upon work supported by the Department of the Army under Air Force Contract No. FA8702-15-D-0001 and the US Department of Defense Air Force Office of Scientific Research, through the Massachusetts Institute of Technology Lincoln Laboratory. The views and conclusions contained herein are those of the authors and should not be interpreted as necessarily representing the official policies or endorsements, either expressed or implied, of the U.S. Government. The #StartSmall foundation provided financial support for this work. Oura Health Oy provided 1400 pieces of hardware and financial support in the form of a sponsored research contract.”

The original Article has been corrected.

